# Effect of a Mediterranean Diet Adapted to the Mexican Population on Indicators of Metabolic Risk in Patients With Obstructive Sleep Apnea: Protocol for a Randomized Controlled Trial

**DOI:** 10.2196/72513

**Published:** 2025-08-21

**Authors:** Gittaim Pammela Torres San Miguel, María de la Luz Sevilla González, Eduardo López Ramírez, María Fernanda Flores López, Lubia Velázquez López

**Affiliations:** 1 Posgrado Facultad de Medicina Instituto Politécnico Nacional Mexico City Mexico; 2 Coordinación Clínica Hospital General de Zona No 8 Instituto Mexicano Del Seguro Social Baja California Mexico; 3 Escuela Superior de Medicina Instituto Politécnico Nacional Mexico City Mexico; 4 Servicio de Geriatría y Clínica del Sueño Hospital Regional No 1 Mexican Social Security Institute Mexico City Mexico; 5 Unidad de Investigación Biomédica Hospital Regional No 1 Mexican Social Security Institute Mexico City Mexico

**Keywords:** obstructive sleep apnea, obesity, hyperglycemia, hypercholesterolemia, Mediterranean diet, nutritional therapy

## Abstract

**Background:**

Obstructive sleep apnea (OSA) is characterized by episodes of intermittent airway obstruction during deep sleep or REM (rapid eye movement). It is associated with cardiometabolic risk diseases. The base treatment is continuous positive airway pressure (CPAP), to which not all patients are able to adapt. The Mediterranean diet (MD) has proven to be effective in reducing cardiovascular risk markers; however, it must be adapted to different populations. Among patients with OSA, it can effectively reduce clinical entity and cardiometabolic risk, while improving quality of life and sleep.

**Objective:**

We aimed to evaluate the effect of an MD adapted to the Mexican diet versus a standard nutritional treatment on metabolic risk indicators in patients with OSA.

**Methods:**

A randomized, 2-arm clinical trial will be conducted with patients with OSA from the Hospital, Mexican Social Security Institute (Instituto Mexicano del Seguro Social) in Mexico. Patients will be randomly included in the group with MD adapted to Mexican foods. With a personalized diet plan adapted from the Mediterranean diet, prototype menus with Mexican foods adapted from the MD will be included for a higher consumption of fruit, vegetables, fiber-rich cereals, and reduction of red meat. In addition to an illustrative plate to improve adherence. The standard diet (SD) group will receive standardized nutritional counseling for patients with OSA. Fasting blood samples will be drawn after 6 and 12 months to identify glucose levels and lipid profiles. Anthropometric and body composition measurements will be taken, and adherence to diet will be recorded after 3, 6, and 12 months. Sleep quality, physical exercise, and life quality will be recorded basally and after 12 months. A multivariate logistic regression analysis will be performed, including the achievement of metabolic indicator control goals, sleep quality, and quality of life as outcome variables. This analysis will be adjusted for other variables that may be statistically significant in the bivariate analysis, such as sex, age, and comorbidities, among others. Statistically significant differences between groups will be considered when the value of *P*<.05.

**Results:**

The protocol was authorized in 2024, and funding is being sought for patient follow-up. All 120 patients (60 per group) will be included in 2025; recruitment will begin in March 2025. The clinical trial is expected to be completed in April 2026.

**Conclusions:**

The results of this study will contribute to evaluate the effect of a nutritional intervention adapted to patients with OSA, seeking to reduce cardiovascular risk indicators in patients, improve their clinical condition, reduce OSA symptoms, and improve patients’ quality of life.

**Trial Registration:**

ClinicalTrials.gov NCT06278571; https://clinicaltrials.gov/study/NCT06278571

**International Registered Report Identifier (IRRID):**

PRR1-10.2196/72513

## Introduction

Obstructive sleep apnea (OSA) is a disease characterized by the partial or total obstruction of airways during sleep [1]. The global prevalence of OSA has been reported to be between 9% and 38%, considering an apnea-hypopnea index (AHI) of ≥5 events per hour; it is higher in men, those with an advanced age, and with urban dwelling [2]. In Mexico, an elevated risk of OSA of 27.3% in adults has been reported, and this risk increases as the BMI is higher. [3]

OSA is estimated to be present in 60% of patients with metabolic syndrome, which can also include, diabetes, obesity, insulin resistance, systemic arterial hypertension, and dyslipidemia [4]. Overweight and obesity contribute to higher amounts of fat in the dilator muscles of neck and tongue, thus increasing OSA. A positive correlation has been found between severe OSA and obesity in adult patients [5].

Daytime symptoms of patients with OSA include excessive daytime drowsiness, complaints of unrefreshing sleep, morning headaches resolved with breathing regulation, irritability, apathy, depression, difficulty to focus, and memory loss, among others [6]. The treatment of choice for patients with OSA, continuous positive airway pressure (CPAP), generates positive air pressure to reduce negative intrathoracic and upper airway pressure, limiting the repetitive hypoxia cycle in the patients; still, adherence is limited [7].

Weight loss in patients with OSA is directly proportional to reduced respiratory events measured by AHI. This was reported in 89 men with OSA who reduced body weight by 7%, fat by 19%, and visceral adipose tissue by 26%, in addition to changes in lifestyle. As a result, OSA was reduced by 15% and 45% of the patients did not need CPAP anymore [8]. Evidence indicates the Mediterranean diet (MD) is widely cardioprotective and contributes to lowering cardiovascular risk and even death [9-11]. The PREDIMED study reported that an MD, mostly consisting of fruit, vegetables, white meat, and dried fruit or olive oil, reduced cardiovascular event symptoms in patients compared to those who followed a controlled diet. The MIMOSA (Mediterranean Diet/Lifestyle Intervention in Obstructive Sleep Apnea) study conducted with 187 adults with OSA showed that an intervention with MD or Mediterranean style had a better effect on improving cardiometabolic indicators than a standard diet (SD) [12]. In addition, the authors report a greater reduction in metabolic syndrome with MD in patients with OSA, even when adjusting fluctuations in body weight as compared with a Mediterranean style or an SD [13,14]. Fewer insomnia episodes have been linked to greater adherence to MD in patients with OSA and insomnia [15]. Although it has been shown to be cardioprotective and improve metabolic indicators, MD must be adapted to other countries. In Mexico, there is scarce evidence on the modification of MD to the Mexican diet and its cardiometabolic effect in patients with OSA. Therefore, the aim of this study is to evaluate the effect of a MD adapted to Mexican eating habits versus a standard nutritional treatment on metabolic risk indicators in patients with OSA.

## Methods

### Study and Population Assignment

A randomized, 2-arm clinical trial will be conducted with patients with OSA from the Regional Hospital, Mexican Social Security Institute (Instituto Mexicano del Seguro Social [IMSS]) in Mexico City with a 12-month follow-up.

The sample size was calculated using the formula for difference in proportions, considering a CI of 95% and a power of 80%. Weight loss is expected in 58% of the sample corresponding to the group given an MD adapted to the Mexican population and in 30% of the group consuming a standard diet, as previously reported in the literature. A sample size of 49 patients per group was obtained and, considering losses of 20%, the total number of patients will be 120 [16].

### Selection Criteria

#### Inclusion Criteria

Men and women aged 30-70 years, diagnosed with moderate and severe OSA will be included. Textbox 1 describes the inclusion and exclusion criteria.

**Selection criteria for patients with obstructive sleep apnea included in the study.**

**Inclusion criteria**

- Men and women
- Aged 30-70 years
- Diagnosed with moderate or severe obstructive sleep apnea
- Use of continuous positive airway pressure (CPAP)

**Exclusion criteria**

- Anatomic alterations of the nose, oropharynx, or maxilla
- Chronic kidney disease under substantive renal function treatment
- Decompensated heart failure
- Any type of cancer
- Neurological disease
- Diagnosis of depression
- Refractory dyslipidemia
- Familial dyslipidemia
- Surgeries in the last 6 months
- BMI>40 kg/m²

#### Study Procedures

The study design is a randomized, 2-arm clinical trial in patients with OSA. The patients will be referred by a specialist from the sleep clinic at the Hospital where the study will take place. Once the inclusion criteria are checked, the study, risks, and benefits will be explained to the patients, questions will be answered by the researchers, and the patients will sign informed consent to participate in the trial.

Software will be used for randomization, and patients will be assigned to either of the following groups: MD with Mexican food and SD.

#### Sociodemographic and Clinical Variables

The medical researcher will conduct a clinical interview to obtain sociodemographic data, pathological background, present comorbidities, and current pharmacological treatment.

A medical questionnaire will be applied by the research team to collect sociodemographic data, pathological-clinical background, and nonpathological information. The patients will answer questions regarding age; clinical history, including pathological and nonpathological background; and pharmacological and nonpharmacological treatments.

#### Arterial Blood Pressure Measurement

Arterial blood pressure will be measured by the medical team participating in the study, using a pregauged blood pressure cuff, and no smoking nor consumption of coffee or cola soft drinks in the previous 30 minutes. The patient will sit in a chair, with the back supported and feet straight on the floor. The left arm will be uncovered and supported on a flat table at heart level. The air chamber (balloon) must cover at least three-fourth of the arm’s length and at least 80% of its circumference.

#### Sleep Variable Measurement

During the first consultation at the Sleep Clinic, measurements of AHI and oxygen desaturation index will be recorded using CPAP, as part of the routine studies patients with OSA undergo. The study takes 7-8 hours.

##### Polysomnography

Polysomnography channels will be set up; they include electroencephalographic derivations and electrooculograms, electromyograms of the chin and legs, respiratory flow signals from the thermistor and nasal cannula, respiratory effort signals, continuous oxygen monitoring, electrocardiogram, and the patient’s body position. The study, lasting 4-6 hours, will be carried out once and is part of a series of tests that patients with OSA undergo.

#### Biochemical Indicator Measurement

Venous blood samples will be taken after fasting for 8-10 hours to measure glucose, lipid profile (cholesterol, triglycerides, high-density-lipoprotein cholesterol, and low-density-lipoprotein cholesterol). Measurements will be recorded during the first consultations and after 6 and 12 months.

#### Anthropometric and Body Composition Measurements

Body composition will be measured by bioimpedance, using an InBody 120 composition analyzer, to obtain fat percentage, fat mass, lean mass, and total body water. Body weight and height will be measured with the same analyzer. The patient must wear as little clothing as possible, without any metal objects (bracelets, earrings, rings, belts, and others). The patient will face forward, place their feet on the metal plates, and extend their arms forward to hold the metal bars at a 90º angle. Height will be measured using an InBody ultrasound stadiometer while the patient is in an upright anatomical position. Anthropometry will be recorded by 2 nutritionists previously standardized, using the method proposed by Habitch and according to the specifications previously [17,18]. The BMI will be obtained from height and weight.

Waist circumference will be measured using a measuring tape halfway between the lowest rib and the top of the right hipbone. The average value of the second and third measurements will be used for analysis. Measurements will be taken during the first consultation and after 3, 6, and 12 months.

#### Sleep Quality Measurement

Sleep quality will be measured using the Epworth Sleepiness Scale (ESS), which evaluates 8 items, each from 0 to 3 points, where 0 indicates the patient would never nod off, 1 indicates a small probability of nodding off or falling asleep, 2 indicates a moderate probability of nodding off or falling asleep, and 3 indicates a high probability of falling asleep. A score from 1 to 6 on the scale is considered regular sleep, one from 7 to 8 points is considered mild sleepiness, and one from 9 to 24 points at a pathological (abnormal) drowsiness [19].

#### Quality of Life Measurement

Quality of life will be measured using a Quebec Sleep Questionnaire (QSQ), which has been validated for the Hispanic population. It contains 32 items focused on measuring daily sleepiness (7,16,20,27), daytime symptoms (1,10,11,14,17,18,19,26,29), nighttime symptoms (4,9,21,25,28,30), emotions (5,6,8,15,24), and social interactions (2,3,12,13). The questionnaire will be applied basally and after 12-month follow-up [20].

#### Physical Activity Measurement

To measure physical activity, the International Physical Activity Questionnaire will be applied. The questionnaire contains 7 items that asses at the physical activity carried out in the past 7 days (hours, minutes, and days of the week). Physical activity will be classified after considering the metabolic equivalent of task values according to the activity (measurement unit of the test). Physical activity is classified in 3 categories: low, moderate, and high (25). It will be measured at the beginning of the trial, and after 6 and 12 months [21].

### Nutritional Intervention Description

#### Group With MD Adjusted Using Mexican Food

The patients in the MD group will be given a normocaloric, personalized diet with the following distribution, 15%-20% protein, 50%-55% carbohydrates, 25%-30% fats, and <7% saturated fats, according to the recommendations for patients with OSA. The Mifflin-St Jeor equation will be applied to calculate the caloric requirements. At the beginning of the intervention, 24-hour reminders will be established to know dietary habits and adjust the diet to the patients’ preferences, customs, and budget. A dietary plan will be designed using equivalent foods. The equivalents will be chosen from the Mexican system of equivalent foods, considering portions, amounts, and preparation of each food. In it, typical foods of the Mexican diet can be found, along with their most common preparation and presentation; for instance: grilled, fried, baked, and uncooked (raw), among others [22].

The MD includes a variety of typical foods from European countries. Therefore, it is necessary to analyze their nutritional characteristics before adapting the diet to Mexican citizens, particularly those patients with OSA taking part in this study. After reviewing the literature, foods belonging to the Mexican diet were identified to show similar characteristics as those in the MD. As part of the adaptation, a comparative chart showing European and Mexican foods was created using the major food groups. Table 1 shows the most representative foods and their adaptation to the Mexican diet, which is explained simply to the patients.

**Table 1:** Mediterranean-Mexican food: proposal for the adaptation of the Mediterranean diet with Mexican food.

Food group/equivalent	Mediterranean diet	Foods in Mexico	Suggested portions and frequency of consumption
Fruit and vegetables	Fruit: grapes, watermelon, strawberries, oranges Vegetables: artichokes, asparagus, lettuce, bell peppers, spinach, broccoli	Fruit: guava, orange, mango, mamey, papaya, blackberry, banana Vegetables: nopal, chayote, cucumber, mushrooms, purslane, quelites, chard, watercress, zucchini, bell pepper, green beans, jicama	Fruit: 1-2 servings per main meal Vegetables: >2 servings per main meal Total: at least 5 servings per day
Healthy Fats	Olive oil, olives, hazelnuts, almonds	Avocado, nuts, peanuts, corn oil, sunflower oil, pumpkin seeds, pistachios, sesame seeds	Olive oil: in every main meal Nuts, seeds, olives, avocado: 1-2 servings per day
Cereals	Whole wheat bread, pasta, rice, couscous	Whole wheat bread, pasta, rice, corn tortilla, oats, amaranth	Bread, pasta, rice, tortilla, oats: 1-2 servings per main meal
Tubers	Potatoes, sweet potatoes	Potatoes, sweet potatoes, yuca	Potatoes, sweet potatoes, yuca: <3 servings per week
Legumes	Lentils, chickpeas, beans, peas	Lentils, beans, chickpeas	Lentils, beans, chickpeas: >2 servings per week
Animal-based foods	Fish and seafood: salmon, cod, squid, mussels, shrimp, oysters Low-fat dairy: yogurt, cheese, milk White meat: chicken, hen, turkey Eggs Red and processed meats: beef, pork, cold cuts	Fish and seafood: tuna, sardines, trout, red snapper, tilapia Low-fat dairy: yogurt, cheese, milk, cottage cheese, curd White meat: chicken, hen, turkey Red and processed meats: beef, pork, cold cuts	Fish: >2 servings per week Low-fat dairy: 2 servings per day White meat: 2 servings per week Eggs: 2-4 servings per week Red meat: <2 servings per week Processed meats: <1 serving per week

This table provides examples of foods characteristic of the general Mexican population. It may vary according to the region of Mexico, seasonality, and accessibility for each patient.

**Figure 1:**
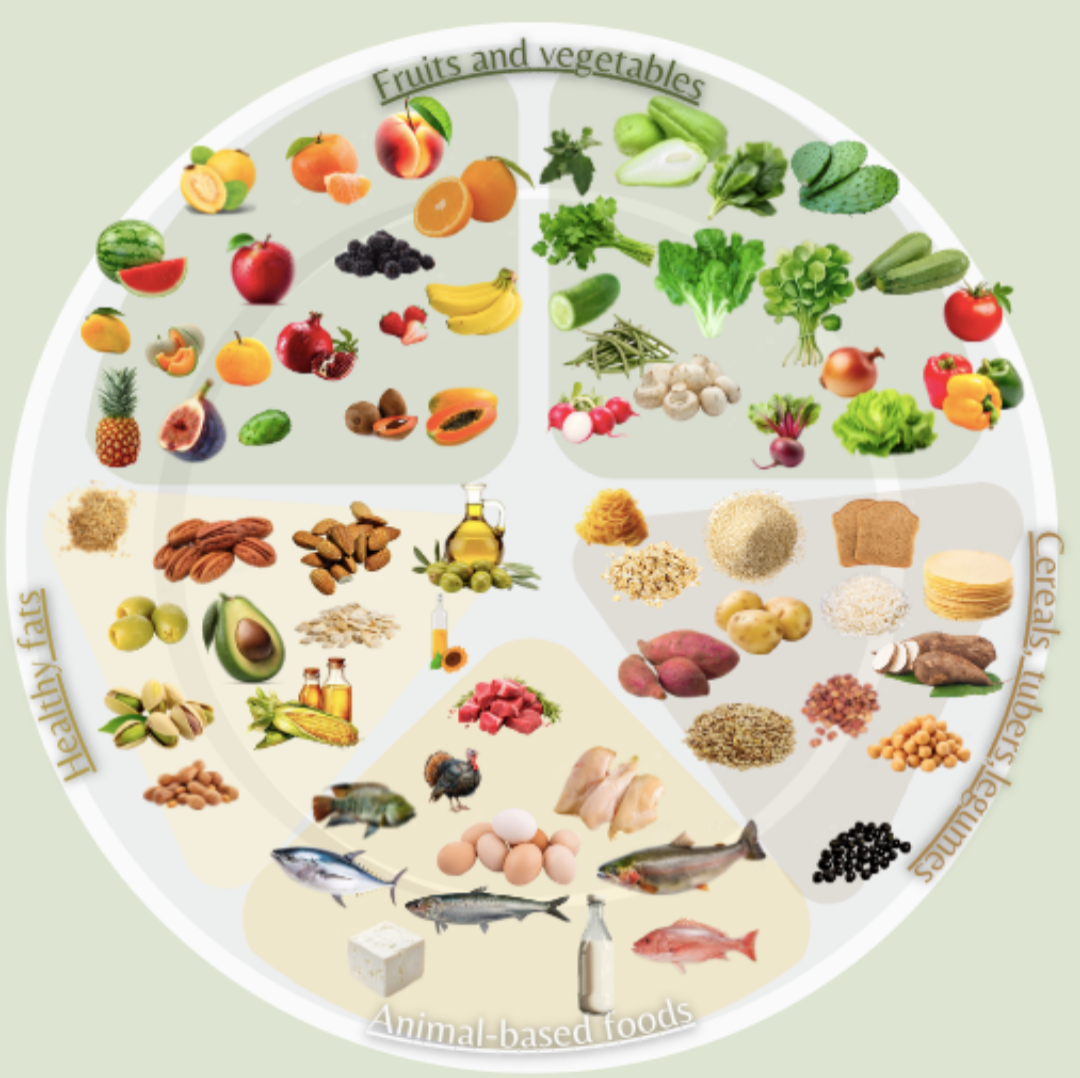
Mediterranean diet plate adapted with Mexican food.

In addition to the equivalency chart including the food groups of both diets, a healthy eating plate MD (see Figure 1), was created as the strategy has been effective in Mexico, where the main foods for a healthy diet are presented in a plate [23]. From this strategy, a plate of MD in Mexico was designed, adapting foods that must be consumed according to the region, culture, preferences, and budget, considering easy access for patients with OSA.

To reinforce the meal plan given to this group, preestablished menus were designed containing foods typical in the MD, such as fruit, vegetables, whole grains, cereals, and white meat. The menus also promoted the intake of vegetable oils and common dried fruit in Mexico. The menus were designed to reach 1200, 1400, 1600, 1800, 2002, and 2200 calories. This group will also continue their common treatment with their physician or group of health care professionals as usual.

#### Assessment of Adherence to the MD With Mexican Food

Adherence to MD will be measured using the 14-point Mediterranean diet adherence screener (MEDAS) questionnaire, consisting of 12 questions on the consumption frequency of foods and 2 questions on intake habits of foods considered part of the MD [24]. In this instrument, each question is scored 0 or 1. One point is given for using olive oil as the main source of fat for cooking, preferring white meat over red meat, or for consuming (1) 4 or more spoons of olive oil per day, (2) 2 or more vegetable portions per day, (3) 3 or more pieces of fruit per day, (4) less than 1 portion of red meat or sausage per day, (5) less than 1 portion of animal fat per day, (6) less than 1 sugary drink per day, (7) 7 or fewer glasses of red wine per week, (8) 3 or more portions of legumes per week, (9) 3 or more portions of fish per week, (10) fewer than 2 cakes or commercial baked goods per week, (11) 3 or more portions of nuts per week, and (12) a plate of traditional tomato, garlic and onion sauce twice or more a week. The total score ranges between 0 and 14 and allows to identify 3 levels of MD, low (0-6), medium (7-8), and high (≥9).

#### SD Group

Patients in the control group will be given a normocaloric diet with the following energy distribution: 15%-20% protein, 50%-55% carbohydrates, 25%-30% fats, and <7% saturated fats, per the guidelines for adult patients with OSA. The Mifflin-St Jeor equation will be applied to identify the caloric requirements of each patient. The energy needs and distribution will be administered as individual nutritional guidance regarding sleep hygiene measures and type of diet for weight loss according to age, sex, current body weight, and present comorbidities. At the beginning of the intervention, 24-hour reminders will be set to know the daily diet. Patients will be given a pamphlet to reinforce healthy eating, and they will continue nutritional consultations with their physicians. This group will also continue the traditional treatment with their physician or group of health care professionals as usual.

#### Follow-Up and Study Procedures

**Figure 2:**
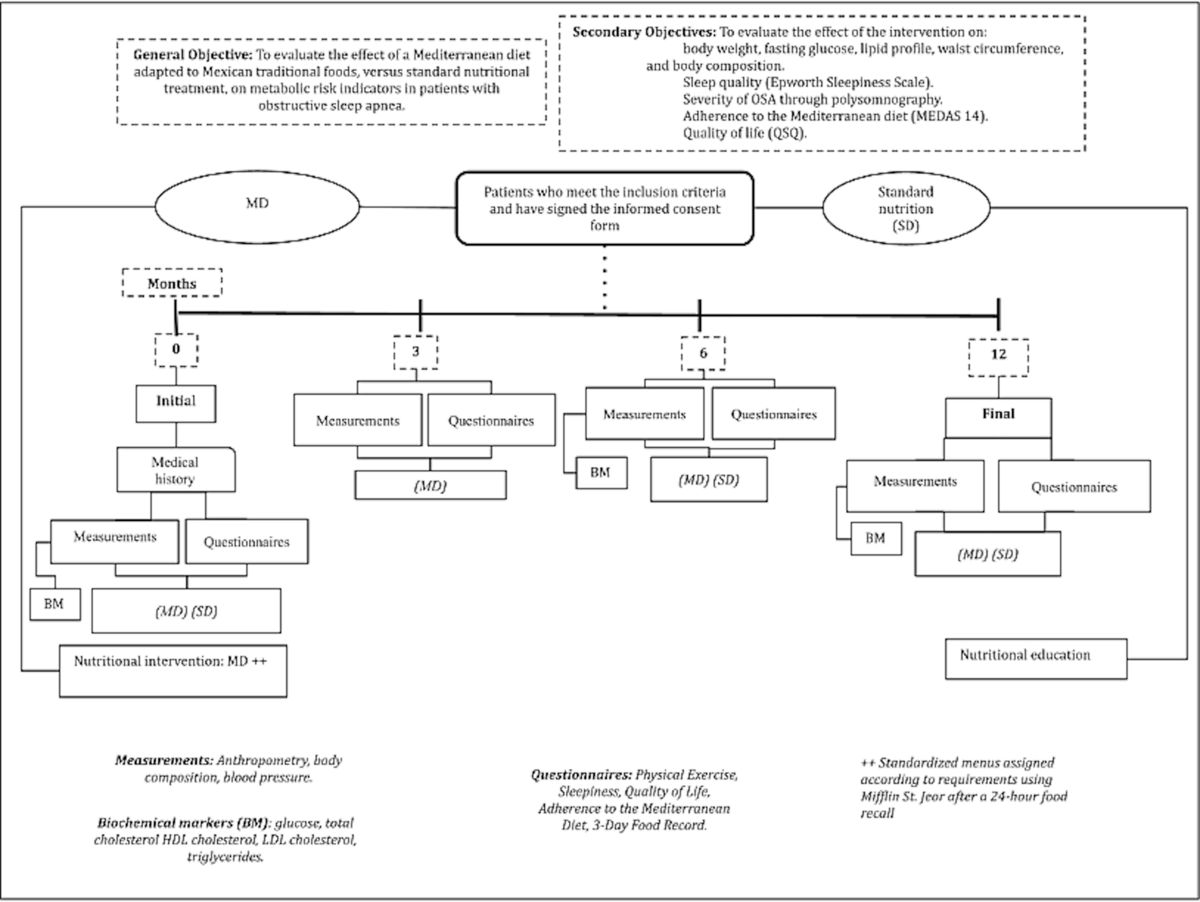
Follow-Up and Study Procedures.

At the beginning of the trial and after 12 months, laboratory data (glucose and lipid profiles), clinical data, arterial blood pressure records, anthropometry, body composition, and dietary records of the previous 3 days will be obtained, along with the quality of life and sleep survey. All the patients will receive the written results and explanation in each clinical consultation, as shown in Figure 2.

Follow-up will continue after 3, 6, and 12 months for MD and 6 and 12 months for SD diet participants, who will attend a consultation with a nutritionist and medical team. In order to measure the adherence to the diet in both groups, the patients will register the 3 days before their clinical visit, 2 registrations from Monday to Friday, and one on weekends. At each visit, nutritional guidelines will be given to improve adherence to the nutritional intervention. The study follow-up is shown in Table 2.

**Table 2:** Study follow-up and procedure in patients with obstructive sleep apnea in both groups.

Time collection	Baseline	3 months	6 months	12 months
Collection of sociodemographic data	MD^a^ and SD^b^	—^c^	—	—
Medical history	MD and SD	—	—	—
Medical evaluation	MD and SD	—	MD and SD	MD and SD
Biochemical data	MD and SD	—	—	MD and SD
Arterial blood pressure and Anthropometric and body composition measurements	MD and SD	MD	MD and SD	MD and SD
Sleepiness with Epworth sleepiness scale	MD and SD	MD and SD	MD and SD	MD and SD
Quality of life with Quebec Sleep Questionnaire (QSQ)	MD and SD	—	—	MD and SD
Physical activity with IPAQ^d^ Questionnaire	MD and SD	—	MD and SD	MD and SD
Adherence to the diet intervention with records of the previous 3 days	MD and SD	MD	MD and SD	MD and SD
Adherence to the diet intervention with MEDAS^e^ Questionnaire	MD and SD	MD	MD and SD	MD and SD
Phone call follow-up	—	SD	—	—

^a^MD: Mediterranean diet.

^b^SD: standard diet.

^c^Not applicable.

^d^IPAQ: International Physical Activity Questionnaire.

^e^MEDAS: 14-point Mediterranean diet adherence screener questionnaire.

### Primary Outcomes

The primary objective of this clinical trial is to evaluate the effect of the MD adapted to the Mexican diet versus an SD treatment on metabolic risk indicators in patients with OSA (body weight, fasting glucose, lipid profile, WC, and body composition).

The secondary outcomes are to evaluate the effect of the intervention on:

1. Sleep quality through ESS.
2. Changes in quality of life through QSQ.
3. Adherence to nutritional therapy intervention through records of meals corresponding to previous and MEDAS questionnaires.

### Statistical Analysis

The statistical analysis of the data will be carried out using SPSS (version 27; IBM Corp). The study population will be described using the background, clinical, and pathological variables to characterize the sample of participants with OSA. The Kolmogorov–Smirnov test will be used to identify the distribution of quantitative variables. For variables with normal distribution measures of central tendency and dispersion will be estimated. For variables with a free distribution mean and IQR will be used. Measures of frequency and percentages will be used for sociodemographic and clinical qualitative variables, as well as those of patient proportion with and without glycemic control, and with and without cognitive impairment. A chi-square test will be used to compare the effect of MD versus SD on parameters of control and uncontrol of metabolic risk indicators. Depending on the data distribution, a Student *t* test will be used to compare quantitative variables with parametric distribution between groups, and a Mann-Whitney *U* test will be conducted for free-distribution variables. A multivariate logistic regression analysis will be performed, including the achievement of metabolic indicator control goals, sleep quality, and quality of life as outcome variables. This analysis will be adjusted for other variables that may be statistically significant in the bivariate analysis, such as sex, age, and comorbidities, among others. Statistically significant differences between groups will be considered when the value of *P*<.05.

### Ethical Considerations

This clinical trial was submitted to the Ethics and Research Committee at HGR 1 “Dr Carlos MacGregor Sánchez Navarrro,” IMSS (registry number R-2024-3609-055). The protocol was reviewed twice for the corresponding modifications related to methodological aspects. The researchers will provide each participant with written consent, describing all the procedures of the clinical trial, the risks, and benefits; this consent form clarifies that the patient is free to not participate or to withdraw from the research without affecting their care at the institution. All participant information collected in the research will be kept confidential, and data will be reported anonymously. Participants in this research will not receive any financial compensation.

The protocol was registered at ClinicalTrials.gov (NCT06782737). The aim of the study, risks, and benefits will be explained in detail to the participants who will freely chose to take part in the trial. They will provide written consent describing each procedure in the trial and respect to information privacy. All the researchers participating receive annual training regarding better clinical practices related to research.

## Results

The protocol was approved in 2024, and funding is being sought for patient follow-up, patient inclusion, and execution of RCT in 2025. Currently, 50 patients have been recruited, 28 in the group with a Mediterranean Diet Adjusted Using Mexican Food and 27 in the group with an SD. The data analysis with the expected results will be carried out in February 2026.

It is expected that there will be differences in the intervention groups of patients with OSA enrolled in the study: measurement of blood pressure, measurement of sleep variables, measurement of biochemical indicators, anthropometric and body composition measures, measurement of sleep quality, and measurement of quality of life. We expect to find in patients with MD a higher proportion of cardiometabolic indicators in control, compared with patients in the group with SD. We expect to find differences in multivariate analysis in control goals of cardiometabolic indicators, quality of sleep and quality of life of patients.

## Discussion

### Anticipated Findings

This protocol aims to measure the impact of an MD adapted to the Mexican population on cardiovascular risk indicators, such as glucose, lipid profile, body weight, and blood pressure in patients with OSA. In addition, as secondary objectives we seek to evaluate the effect of the MD on sleep quality and quality of life, as well as the improvement in diet quality.

There is enough evidence on the cardioprotective effect of MD, which is not only proven in European countries: there are clinical trials evaluating MD adapted to the Chilean population suffering metabolic syndrome [25]. It has been proven that interventions aimed at lifestyle improve AHI, showing a reduction by –4.55 events per hour and improving weight and BMI among patients with OSA; however, no effect on sleep and life quality has been found [26]. Despite the ample evidence supporting the cardioprotective benefits of the MD, its evaluation has been predominantly focused on patients with obesity, diabetes, and, to a lesser extent, patients with OSA. The impact of adapting the MD diet with food in Mexico has been examined to a lesser extent.

The relationship between cardiometabolic risk factors and sleep duration has been previously reported in 47 patients with OSA in a rural community in the southeastern United States. The authors reported that shorter sleep duration was associated with an increased risk of hypertension, obesity, and impaired metabolic indicators. [27]. The results of this study suggest that an MD intervention with a preference for fruit, vegetables, high-fiber cereals, and white meat protein may improve cardiometabolic indicators.

Obesity and related diseases, like diabetes, hypertension, and dyslipidemia, represent a greater risk for patients with OSA. Then, CPAP treatment combined with weight loss is an effective strategy to reduce cardiovascular risk [28]. Adaptation to the patient's tastes, customs, and budget is necessary to improve adherence to MD. This study seeks to adequate MD to the Mexican diet to make it accessible, since typical MD includes foods considered high-priced for the population receiving care at public health care institutions in Mexico.

Furthermore, improvement in cardiometabolic indicators, such as glucose, lipid profile, and weight loss, is likely. Patients are expected to lose 5%-10% of their initial body weight, as reported by similar interventions [12].

Sleep quality in patients with OSA could improve after the proposed nutritional intervention, while the number of obstructive respiratory events could be reduced given that the size of the oropharyngeal lumen is reduced because of body fat loss [29]. Besides the reduction in obesity, the diet is expected to improve fasting glucose, arterial blood pressure, and dyslipidemia in patients with OSA taking part in the trial. We also expect this trial to positively affect daytime drowsiness in the patients and improve their quality of life. This clinical trial would be one of the few conducted in Mexico with patients with OSA where the implementation of low-cost strategies aims at improving cardiometabolic indicators and quality of life of those patients with OSA who receive care at public health care institutions in Mexico.

Among the limitations of the present study, adherence to the MD could be found; therefore, it is relevant to adapt the diet to the economic resources, habits, and customs, which allows an adequate adherence to the MD. A personalized diet and a timely follow-up will be provided to reinforce adherence to the nutritional intervention. It has also been shown that the use of CPAP is limited in the population with OSA, since they do not consider it necessary for their condition or it is difficult to use it to sleep adequately [30]. One of the potential limitations of the study is the use of a self-reported tool to collect sleep quality information. There will be limitations to the generalizability of the effect of the intervention, as it will be a single-center study. In this sense, as one of the main variables is to measure the improvement in sleep quality, this could be improved with the use of CPAP, so its use will be recorded in the follow-up visits, to include it in the multivariate analysis.

### Conclusion

This study seeks to contribute to improving the diet of patients with OSA, thus enhancing the cardiometabolic risk indicators and reducing the patients’ risk of cardiovascular diseases. In addition, we expect to improve the patients’ sleep quality and lifestyle. We consider this intervention will be useful to create a proposal including an MD adapted to Mexican habits that improve the dietary needs of patients with OSA and similarly those of patients with diabetes, obesity, hypertension, and other comorbidities.

## Appendix

Multimedia Appendix 1 SPIRIT guidelines.

Multimedia Appendix 2 Peer review report by the Research Committee of the Hospital Sede Instituto Mexicano del Seguro Social.

## Abbreviations

AHI: apnea-hypopnea index
CPAP: Continuous positive airway pressure
ESS: Epworth Sleepiness Scale
IMSS: Instituto Mexicano del Seguro Social
MD: Mediterranean diet
MEDAS: Mediterranean diet adherence screener
MIMOSA: Mediterranean Diet/Lifestyle Intervention in Obstructive Sleep Apnea
OSA: obstructive sleep apnea
QSQ: Quebec Sleep Questionnaire
SD: standard diet

## Data Availability

The datasets generated or analyzed during this study are not publicly available due (the data is confidential) but are available from the corresponding author on reasonable request.
