# Supplementary material for: Effect of a Mediterranean Diet Adapted to the Mexican Population on Indicators of Metabolic Risk in Patients With Obstructive Sleep Apnea: Protocol for a Randomized Controlled Trial
Source: JMIR Res Protoc. 2025 Aug 21;14:e72513. doi: 10.2196/72513 (PMC12411790; doi:10.2196/72513)
Supplement: Multimedia Appendix 2 [file resprot_v14i1e72513_app2.pdf]

Peer review process by the research committee of the Hospital sede Instituto Mexicano del Seguro Social.

Research protocol: EFECTO DE LA DIETA TIPO MEDITERRÁNEA VERSUS DIETA ESTÁNDAR EN INDICADORES CARDIOMETABÓLICOS DE PACIENTES CON APNEA OBSTRUCTIVA DEL SUEÑO

Regresar

Protocolo de Investigación

Título del protocolo

EFECTO DE LA DIETA TIPO MEDITERRÁNEA VERSUS DIETA ESTÁNDAR EN INDICADORES CARDIOMETABÓLICOS DE PACIENTES CON APNEA OBSTRUCTIVA DEL SUEÑO

Investigador responsable

Velázquez López Lubia

Adscripción

Unidad de Investigación en Epidemiología Clínica, HGR1 Carlos McGregor (UNIDAD DE INVESTIGACION BIOMEDICA)

Estatus: Dictaminado
Comité: 3609
Folio: F-2024-3609-034
Dictamen: **Aprobado**
Número de registro: R-2024-3609-055

Tema prioritario asignado (Secretario): Enfermedades respiratorias crónicas, Enfermedades respiratorias crónicas
Nivel de prevención asignado (Secretario): Prevención Primaria

Resumen del protocolo

Marco teorico:
La apnea obstructiva del sueño (AOS) se calcula a nivel mundial en 1 de cada 7 individuos. Los pacientes con AOS Tienen una inflamación crónica de bajo grado se relaciona con arterioesclerosis. El efecto de la dieta mediterránea en indicadores de riesgo metabólico aun no es concluyente en población con AOS.

Objetivos:
General:
Evaluar el efecto de una dieta mediterránea adaptada a la alimentación mexicana, versus tratamiento nutricional estándar, en indicadores de riesgo metabólico de pacientes con Apnea obstructiva del sueño

Observaciones al protocolo

Estimado Investigador, posterior a sesionar su protocolo el Comité Local de Investigación en Salud lo ha APROBADO, considerando: cambiar el texto del glosario que aparece en inglés al español, revisar el orden de las citas bibliográficas, la operacionalización de la variable "intervención" debe ser más clara, verificar la congruencia de los criterios de inclusión con la primera parte del instrumento de recolección de datos.

Evaluación No. 2 del comité de INVESTIGACIÓN

Dictamen del comité:

Aprobado

Observaciones:
Estimado Investigador, posterior a sesionar su protocolo el Comité Local de Investigación en Salud lo ha APROBADO, considerando: cambiar el texto del glosario que aparece en inglés al español, revisar el orden de las citas bibliográficas, la operacionalización de la variable "intervención" debe ser más clara, verificar la congruencia de los criterios de inclusión con la primera parte del instrumento de recolección de datos.

Fecha de reunión
2024-09-30

Fecha Envío	Fecha Dictamen	Acta de Dictamen
2024-08-09	2024-10-02	**Descargar dictamen**

Nombre	Evaluación	Revisión No.
Revisor 1	**Ver revisión**	2
Revisor 2	**Ver revisión**	2

Evaluación No. 1 del comité de INVESTIGACIÓN

Dictamen del comité:

Modificar y volver a presentar

Observaciones:
Estimado Investigador, posterior a sesionar su protocolo el Comité Local de Investigación en Salud dictamina que debe MODIFICAR Y VOLVER A PRESENTAR considerando: 1. Resaltar los cambios con marcatextos amarillo, 2. Presentar los resultados y datos que sustentan el beneficio de la dieta mediterránea en pacientes con AOS, 3. Revisar ortografía y sintaxis en todo el documento, 4. Revisar el orden de las referencias bibliográficas, apego a las normas Vancouver y que todos las referencias que se citan aparezcan en la bibliografía, 5. Verificar que las palabras clave estén indexadas en el DECS/MESH, 6. Detallar cuál es la dieta mediterránea adaptada a la alimentación mexicana y el sustento o validación que tiene, 7. Incluir la referencia en la hipótesis alterna para el porcentaje de reducción esperado, 8. Verificar congruencia de hipótesis nula y alterna, 9. Justificar porqué solo se considera a la población de una unidad médica, 10. Modificar la redacción de los criterios de inclusión de forma que sean claros "con o sin diabetes" o "con o sin hipertensión" , 11. Sustentar en el marco teórico todas las variables de estudio, 12. Justificar porqué se toma como referencia el 70% de adherencia a la dieta mediterránea, 13. Corregir la definición conceptual de la variable "sexo", género no es lo mismo que sexo, 14. Corregir la clasificación de la variable edad de acuerdo a la unidad de medición, 15. Solo se consideran dos opciones para la variable estado civil, considerar que existen más valores posibles, 16. No es clara la definición conceptual de la variable "evolución de la enfermedad", 17. La variable calidad de vida debe sustentarse en el marco teórico, 18. Indicar en el diseño estadístico cómo se identificará la distribución de variables numéricas, 19. Detallar quienes realizarán las mediciones, para controlar sesgos relacionados con mediciones por distintas personas, 20. En los criterios de exclusión, aparecen comorbilidades y la frase "entre otras" que no es clara, debe delimitarse cuales son las otras enfermedades, 21. No se detalla en la descripción general del estudio qué instrumento validado se empleará para medir el ejercicio físico, 22. Diferenciar mediante símbolos o colores las actividades programadas y las realizadas en el cronograma. Realizar modificaciones y enviar a la brevedad.

Documentos del protocolo

Nombre	Tipo de archivo	Fecha
**PI-2024-3334-CRONOGRAMA.pdf**	Cronograma de actividades	2024-08-09 12:24:19
**PI-2024-3334-PROTOCOLO.pdf**	Protocolo	2024-08-09 12:23:48
**PI-2024-3334-INSTRUMENTOS-1.pdf**	Instrumentos de recolección	2024-07-09 11:42:45
**PI-2024-3334-CONSENTIMIENTO.pdf**	Carta de consentimiento informado	2024-07-09 11:42:08
**PI-2024-3334-INSTRUMENTOS.pdf**	Instrumentos de recolección	2024-04-18 10:44:38
**PI-2024-3334-NO-INCONVENIENCIA-DIR.pdf**	Carta de no inconveniencia del director de la unidad	2024-04-18 10:44:27
Nombre	Tipo de archivo	Fecha

Documentos cargados por el comité

Nombre	Tipo de archivo	Fecha
**PI-2024-3334-DICTAMEN.pdf**	Dictamen	2024-06-12 14:25:42
**PI-2024-3334-DICTAMEN-1.pdf**	Dictamen	2024-10-02 15:13:27
Nombre	Tipo de archivo	Fecha

Revisores del protocolo

Nombre	Fecha de asignación	Fecha de respuesta	Revisión No.
**Revisor 1**	2024-09-10 08:55:00	2024-09-17	2
**Revisor 2**	2024-09-10 08:55:07	2024-09-14	2
**Revisor 2**	2024-04-25 11:20:27	2024-04-28	1
**Revisor 1**	2024-05-30 07:55:13	2024-06-03	1

Evaluación No. 2 del comité de INVESTIGACIÓN

Dictamen del comité:

Evaluación No. 3 del comité de Ética en Investigación

Dictamen del comité:

Aprobado

Observaciones:
Estimada Investigadora, el Comité de Ética en Investigación ha considerado en su sesión ordinaria que su protocolo cumple con los aspectos éticos y este fue aprobado.

Fecha de reunión
2024-09-18

Fecha Envío	Fecha Dictamen	Acta de Dictamen
2024-09-10	2024-09-19	**Descargar dictamen**

Nombre	Evaluación	Revisión No.
Revisor 1	**Ver revisión**	3
Revisor 2	**Ver revisión**	3

Evaluación No. 2 del comité de Ética en Investigación

Dictamen del comité:

Aprobado

Observaciones:
Estimada Investigadora, el Comité de Ética en Investigación ha dictaminado que su protocolo cumple con los aspectos éticos y puede ser aprobado en su versión actual.

Fecha de reunión
2024-05-24

Fecha Envío	Fecha Dictamen	Acta de Dictamen
2024-05-17	2024-05-24	**Descargar dictamen**

Nombre	Evaluación	Revisión No.
Revisor 1	**Ver revisión**	2
Revisor 2	**Ver revisión**	2

Evaluación No. 1 del comité de Ética en Investigación

Dictamen del comité:

Modificar y volver a presentar

REVISOR 1.

Evaluación del protocolo

EFECTO DE LA DIETA TIPO MEDITERRÁNEA VERSUS DIETA ESTÁNDAR EN INDICADORES CARDIOMETABÓLICOS DE PACIENTES CON APNEA OBSTRUCTIVA DEL SUEÑO

1. Título

1.1. ¿Se ajusta a las características del trabajo propuesto?

● Si
○ No

2. Antecedentes científicos

2.1 ¿La información se relaciona en forma directa con el problema en estudio?

● Si
○ No

2.2 ¿Se fundamenta de manera adecuada el planteamiento del problema, los objetivos y los métodos?

Planteamiento: ○ Si ● No
Objetivos: ● Si ○ No
Métodos : ○ Si ● No

2.3 ¿Las referencias bibliográficas están bien señaladas y concuerdan con las afirmaciones que se les atribuye?

● Si
○ No

3. Planteamiento del problema

3.1. ¿Identifica claramente el problema y lo aísla de otros similares?

● Si
○ No

3.2. ¿En él se identifican las variables en estudio y la relación entre ellas?

○ Si
● No

4. Objetivo

4.1 ¿Cada objetivo (general y/o específico) está planteado de manera que permita diseñar un estudio para lograr su consecución?

Objetivos generales: ● Si ○ No
Objetivos específicos: ● Si ○ No ○ No aplica

4.2. ¿Cada objetivo (general y/o específico) está adecuadamente operacionalizado?

Objetivos generales: ● Si ○ No
Objetivos específicos: ● Si ○ No ○ No aplica

5. Hipótesis de Trabajo

5.1. ¿Hay un objetivo por cada hipótesis que se planteó?

● Si
○ No

5.2. ¿Las hipótesis predicen lógicamente la respuesta probable a las preguntas que se formularon en el planteamiento del problema?

● Si
○ No

5.3. ¿Se identifica la dirección y el compromiso de cada hipótesis?

● Si
○ No

6. Programa de Trabajo

6.1. ¿Está bien caracterizado el universo de trabajo y la propuesta para obtener la muestra necesaria y adecuada para el estudio?

6.2. ¿Se describen los criterios de selección?

◉ Si
○ No

6.3. ¿Los criterios de selección son adecuados para el estudio?

○ Si
◉ No

6.4. ¿La definición de las variables tanto la conceptual como la operacional (cómo se medirán), es clara y adecuada?

◉ Si
○ No

¿Cómo se medirán?

Formato de captura

6.5 ¿La reproducibilidad y validez de los métodos y/o instrumentos de medición que se utilizarán en el estudio está descrita y es adecuada?

◉ Si
○ No

6.6 ¿La descripción del estudio es clara y está suficientemente detallada?

◉ Si
○ No

6.7 ¿La sistematización de la recolección de los datos es adecuada?

◉ Si
○ No

6.8 La descripción de los procedimientos, tanto observacionales como experimentales, es clara y está suficientemente detallada?

○ Si
◉ No

6.9 ¿El estudio incluye alguna forma de control de calidad?

◉ Si
○ No

6.10 ¿Se describen los métodos para procesar los datos y, en su caso, la propuesta para el análisis estadístico que se planea utilizar?

◉ Si
○ No

6.11 ¿En caso de ser pertinente el análisis estadístico, él que se propone es acorde a la forma en que se calculó el tamaño de la muestra?

◉ Si
○ No

7. Congruencia

7.1. ¿Existe congruencia entre la pregunta, el diseño y la descripción del estudio?

◉ Si
○ No

7.2. ¿Los objetivos y la metodología permitirán contestar la pregunta planteada?

◉ Si
○ No

8. Originalidad

8. Originalidad

8.1 ¿Aporta algo nuevo a nivel internacional o nacional?

Nacional: ◉ Si ○ No
Internacional: ◉ Si ○ No ○ No aplica

9. Trascendencia

9.1. ¿El trabajo es importante en su campo de especialidad, de acuerdo con su aportación al avance del conocimiento, aplicabilidad, impacto potencial, consecuencias, etc.?

Avance del conocimiento ◉ Si ○ No
Aplicabilidad: ◉ Si ○ No
Impacto potencial: ◉ Si ○ No

Especificar

El estudio puede ser valioso si se encuentra mejoría en los apcientes que reciben la dieta mediterranea

10. Aspectos éticos

10.1. ¿Requiere carta de consentimiento informado?

◉ Si
○ No

10.2. ¿Presenta la carta de consentimiento informado?

◉ Si
○ No
○ No aplica

10.3. ¿La carta de consentimiento informado es adecuada y completa para el estudio?

◉ Si
○ No
○ No aplica

10.4. ¿El lenguaje de la carta de consentimiento informado es accesible para el paciente?

◉ Si
○ No
○ No aplica

10.5. ¿La redacción de la carta de consentimiento informado manipula la decisión del paciente?

◉ Si
○ No
○ No aplica

10.6. ¿En la carta se describen con claridad los riesgos y beneficios para los participantes?

◉ Si
○ No
○ No aplica

10.7. ¿El protocolo considera una adecuada evaluación de los riesgos y beneficios de su realización?

◉ Si
○ No

10.8. ¿La selección de los sujetos de estudio es imparcial, sin sesgo social, racial, sexual y/o cultural?

◉ Si
○ No

11. Tipo de Investigación que se propone

11. Tipo de Investigación que se propone

○ Biomédica
◉ Clínica
○ Epidemiológica
○ Sistemas de la salud
○ Educación
○ Economía de salud
○ Otros

12. Aspectos subsidiarios

12.1. ¿La sintaxis y la ortografía son correctas?

○ Si
◉ No

12.2. ¿Otorga los créditos en forma adecuada?

◉ Si
○ No

Recomendaciones y Comentarios ANÓNIMOS para el autor

Cambiar el glosario a idioma español

APROBADO CON MODIFICACIONES

REVISOR 2

Recomendaciones y Comentarios ANÓNIMOS para el autor

Se realizaron los cambios solicitados en la evaluación previa de manera satisfactoria
